# Rotation‐Based Snap‐Fit Mechanical Metamaterials

**DOI:** 10.1002/advs.202501749

**Published:** 2025-03-24

**Authors:** Rui Xu, Yulong He, Chuanqing Chen, Jiapeng Sun, Xin Li, Ming‐Hui Lu, Yan‐Feng Chen

**Affiliations:** ^1^ College of Engineering and Applied Sciences Nanjing University Nanjing Jiangsu 210093 P. R. China; ^2^ School of Mechanical Engineering Nanjing University of Science and Technology Nanjing 210094 P. R. China

**Keywords:** cross‐dimensional assembly, energy absorption, multistable mechanical metamaterials, robotic grippers, rotational snap‐fit structures

## Abstract

Multistable mechanical metamaterials have broad application prospects in various fields due to their unique configuration transformation ability, such as energy absorption, shape reconstruction, soft actuator design, mechanical storage, and logic operation. Currently, the steady‐state transition mechanisms for most multistable mechanical metamaterials rely on translational displacement input, while the rotational input mechanisms are rarely studied. Here, a curved beam snap‐fit structure is proposed to realize the multistable transition of metamaterials under rotational load. Their mechanical characteristics and influencing factors are discussed in detail through theoretical analysis, numerical simulation, and experimental verification. In addition, related rotational multistable mechanical metamaterials prototypes are designed. Their potential applications in the fields of energy absorption or robotics are demonstrated, which opens up new ideas and directions for the multifunctional applications of mechanical metamaterials.

## Graphical abstract

This article introduces a rotational snap‐fit structure composed of a circular cantilever beam snap‐fit and a circular groove. It demonstrates multistability during the rotation process. This innovative design has potential applications in various fields, such as mechanical metamaterials, shape deformation, and robotic grippers, showcasing its versatility and practical value.

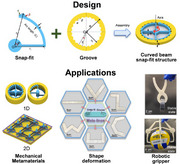

## 1 Introduction

Metamaterials are artificially designed materials with extraordinary properties that depend on the designed structure rather than the inherent properties of the material,^[^ ^1^, ^2^, ^3^ ^]^ and have been widely used in multiple disciplines such as electromagnetism,^[^ ^4^ ^]^ optics,^[^ ^5^ ^]^ thermology,^[^ ^6^ ^]^ acoustics,^[^ ^7^ ^]^ and mechanics.^[^ ^8^ ^]^ In particular, mechanical metamaterials (MMs), as an emerging field, show extraordinary properties such as negative Poisson's ratio,^[^ ^9^ ^]^ multistability^[^ ^10^ ^]^ and negative thermal expansion,^[^ ^11^ ^]^ and have a promising future in the fields of aerospace, biomedicine, and intelligent structures.^[^ ^8^, ^12^, ^13^, ^14^ ^]^

As an important branch of MMs, multistable mechanical metamaterials (MMMs) are capable of forming and stably maintaining several different configurations under external loads.^[^ ^12^, ^15^, ^16^ ^]^ They are usually composed of basic units with bistable properties, and these units can realize reversible switching between states by snap‐through deformation, which makes them have potential applications in many fields. In the realm of energy absorption,^[^ ^16^, ^17^, ^18^, ^19^ ^]^ they can effectively absorb and dissipate external shock energy by means of configuration transformation, thus enhancing system safety. In the context of shape reconfiguration,^[^ ^20^, ^21^, ^22^ ^]^ it can control the shape of materials and provide new ideas for adaptive structural adjustment. Furthermore, in the design of soft actuators and soft robotics,^[^ ^23^, ^24^, ^25^ ^]^ MMMs can provide various motion modes and high flexibility. Additionally, in mechanical storage and logic operations,^[^ ^26^, ^27^, ^28^ ^]^ the reversible multistability of these materials opens new possibilities for information storage and processing, potentially enabling data storage solutions in extreme environments.

At present, the steady‐state transition mechanism of most MMMs depends on the displacement input in the form of compression or tension (i.e., translation). In microscale applications, MMs based on bistable curved beam structures have been used to grasp and release microscopic objects,^[^ ^29^ ^]^ utilizing the snap‐through deformation properties of the material under compressive loads to provide effective mechanical means for precision manipulation. Moreover, there is also an X‐shaped scalable tri‐stable unit designed through the innovation of kirigami microstructure technology, which constructs hierarchical MMs,^[^ ^26^ ^]^ showing ternary logic operation and amplitude modulation ability, and all of them realize the structural steady state transformation through the input of compressive and tensile displacements.

There are relatively few existing studies on MMMs in the form of rotation as an input mechanism. Rotation, as the basic mechanical form of an object's circular motion around a center,^[^ ^30^, ^31^, ^32^ ^]^ is widely found in nature and engineering, such as the rotation of the earth, gears, and wheels rotation. In the scenario of rotary switch, mechanical combination lock, etc., it is necessary to realize multistable structure switching by torsion. These materials can be triggered by rotating inputs to achieve stable configuration reciprocal switching, enriching functions, and expanding engineering applications, such as precision mechanical design, automation control, and safety protection.

Some folding and expandable origami/kirigami structures with rotational symmetry, such as Kresling origami,^[^ ^33^ ^]^ Square‐twist origami,^[^ ^34^ ^]^ rotating squares kirigami,^[^ ^35^ ^]^ etc., have been widely used in medical and aerospace engineering fields, such as medical stent deployment, solar panel deployment.^[^ ^36^ ^]^ Taking the Kresling origami structural unit as an example, it can not only stretch, bend, and deform,^[^ ^37^ ^]^ but also show outstanding flexibility and grasping ability under the precise control of an external magnetic field after assembling into a soft and flexible robotic arm, which can be used for precision operation and object manipulation. However, when existing origami/kirigami structures realize multistable configuration transformation, it often involves the coupling of multiple forms of motion, such as stretching and twisting, which limits the degree of freedom of motion, especially in the case of purely rotational motion, and affects the performance of specific application scenarios.

Although pure torsional motion is common in mechanical systems, there are relatively few research reports on MMMs under pure torsional input mode, especially those involving cyclic rotation.^[^ ^38^ ^]^ Currently, there are some rotation‐driven MMMs, but most of them provide a fixed number of stable states,^[^ ^39^, ^40^ ^]^ that is, a finite number of stable states. In addition, inspired by previous research on the construction of bistable basic units with two magnets arranged in parallel, the development of rotating multistable structures is achieved by the circular arrangement of permanent magnets and rotating magnet pairs, which can store mechanical energy and release it as rotating waves.^[^ ^41^ ^]^ However, the structure depends on magnets, and the magnetic field regulation system is complicated, which limits the practical application. The complex structure and multi‐component precision assembly increase the difficulty of manufacturing. Due to magnetic field interaction, miniaturization faces challenges, which limits its application in precision devices and MEMS.

In order to overcome the above difficulties, this study, based on the previous design of the straight beam snap structure, evolved into the arc‐shaped curved beam snap‐fit and arc‐shaped groove, i.e., rotation‐based snap‐fit mechanical metamaterials (RSMMs). Through the effective assembly of the snap‐fit and groove, a multistable mechanism with torsion as the input form is achieved. The metamaterials are able to stably maintain multiple configurations and switch between them when subjected to rotational loads. A combination of theoretical analysis, numerical analysis, and experimental verification is used to investigate and confirm the multistable mechanical properties, and the multiple factors affecting the properties, such as the geometry and number of clips, symmetry, and the length of the cantilever beam, are explored in detail. We have also designed 1D and 2D RSMMs by arranging snap‐fit and groove structures in a stacked or tiled manner. In addition, we propose the concept of 1D meta‐lines, which demonstrates the remarkable cross‐dimensional assembly capability and the ability to assemble various 1, 2, and even 3D graphic structures. Finally, we demonstrate the potential applications of RSMMs in energy absorption and robotics, opening up new ideas and directions for the multifunctional applications of MMs.

## 2 Results and Discussion

### 2.1 Design and Mechanical Response of the Rotational Snap‐Fit Structure

**Figure 1:**
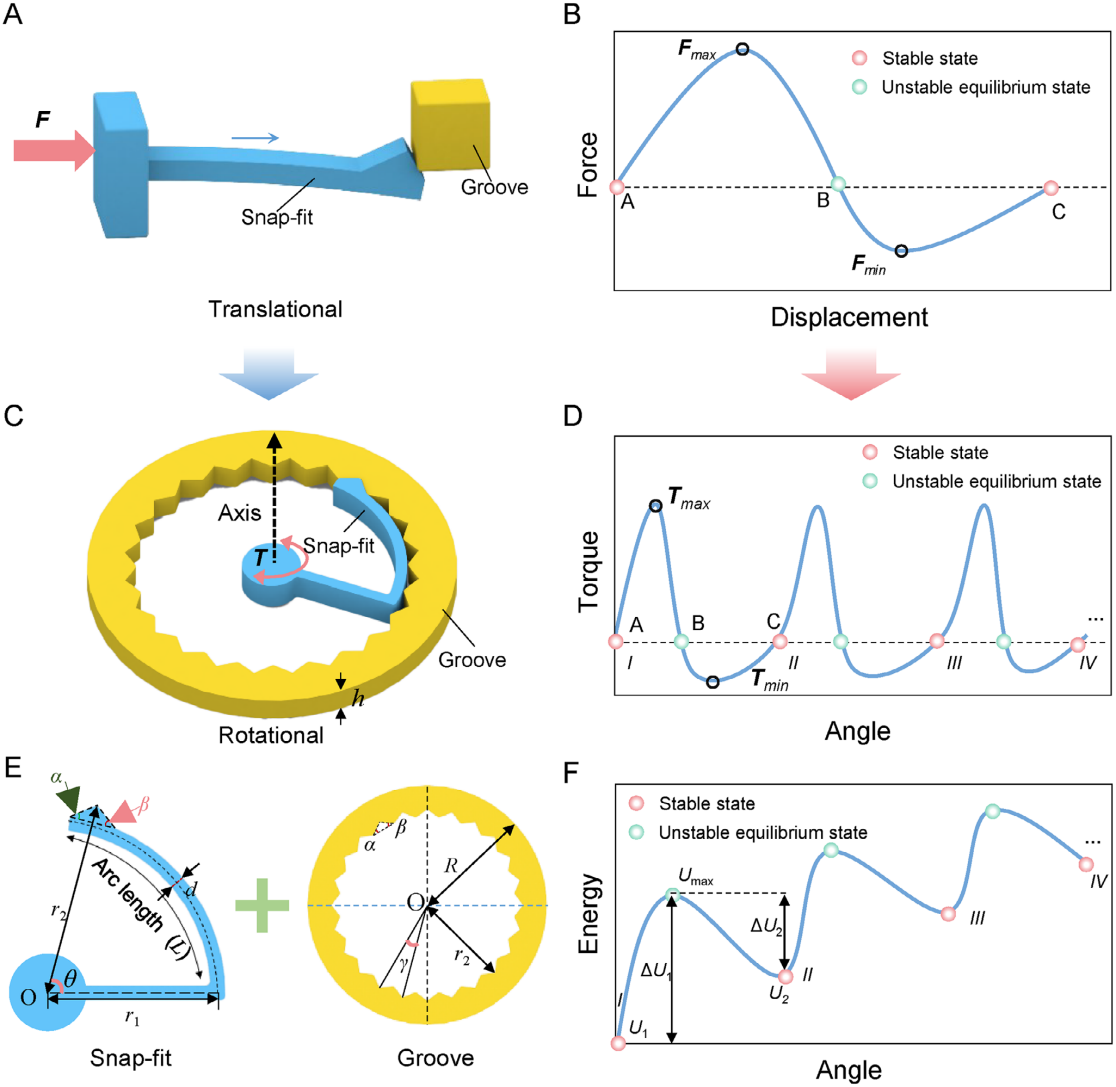
Snap‐fit unit design and mechanical properties. A) Structure of the translational snap‐fit unit: it contains a bendable cantilever snap‐fit and a groove. B) Mechanical response of the translational snap‐fit assembly. C) Rotational snap‐fit structure: consisting of curved cantilever beam snap‐fit and groove. D) Mechanical response of rotational snap‐fit assembly. E) Detailed dimensioning of the rotational snap‐fit, covering the detailed parameters of the snap‐fit and groove. F) Energy landscape of rotational snap‐fit assembly.

In contemporary industrial design, snap‐fit structures are widely used in assembling electrical appliances, automobiles, toys, and various housings due to their excellent functionality and cost‐effectiveness. These structures work by the precise interlocking of the snap‐fit and groove to form a secure connection (**Figure** **1**A). The intersection of materials science and mechanical engineering has made the mechanical behavior of snap‐fit structures a key research focus, especially in energy absorption and soft robotics.^[^ ^16^ ^]^ During assembly, the snap‐fits cantilever beam undergoes elastic deformation and returns to its original shape after assembly. This requires overcoming the critical assembly force (*F*_max_) and transitioning from the initial state (A) to an unstable equilibrium (B) and then to the final assembled state (C), demonstrating the bistable mechanical response of the snap‐fit structure (Figure 1B). Detailed analyses of this process have been presented in previous works^[^ ^16^, ^42^, ^43^ ^]^ and will not be repeated here.

Based on the traditional straight‐beam snap‐fit structure, this study proposes a novel snap‐fit design incorporating a circular groove structure, namely the rotational snap‐fit structure, which capitalizes on the unique properties of circular cantilever beams. Its configuration is shown in Figure 1C. This innovative design overcomes the limitations of conventional straight‐beam snap‐fits, introducing an alteration in the mechanical behavior under rotational stress through the utilization of circular cantilever beam snap‐fit and groove combinations. As depicted in Figure 1C, the blue circular cantilever beam snap‐fit corresponds to the straight cantilever beam snap‐fit in Figure 1A, and likewise, the yellow groove part exhibits a similar correspondence. Figure 1D presents the torque‐angle curve of the rotational snap‐fit structure during torsion. This curve demonstrates a striking similarity between the torque‐angle relationship in rotation and the force‐displacement relationship in straight‐beam snap‐fits during translational assembly, manifesting typical multistable traits. Notably, as the rotational angle augments, the torque values oscillate from positive to negative and back to positive, with corresponding maxima (*T*_max_) and minima (*T*_min_). Moreover, the torque‐angle curve exhibits two stable equilibrium points (point A and point C) and an unstable equilibrium point (point B). The rotational stiffness, defined as the derivative of torque with respect to the rotational angle, remains positive as the angle rises until the torque reaches *T*_max_, after which it turns negative until *T*_min_ is reached. This phenomenon verifies that the rotational snap‐fit structure embodies the multistability characteristic of circular cantilever beams.

Moreover, this study identifies that the energy‐angle curve for the rotational snap‐fit structure mirrors the energy‐displacement curve of the straight‐beam snap‐fit structure. As the rotational angle increases, the system's energy first rises and then decreases, a trend illustrated in Figure 1F. At the stable equilibrium points A and C, the system energy reaches local minima (*U*_1_ and *U*_2_), while the energy attains a maximum value *E*_max_ at the unstable equilibrium point B. The transition between steady states necessitates overcoming energy barriers Δ*U*_1_ or Δ*U*_2_ (Δ*U*_1_ = *E*_max_ – U_1_, Δ*U*_2_ = *E*_max_ – *U*_2_). Although the rotational snap‐fit structure shares several similarities with the straight‐beam snap‐fit, its capacity for continuous rotation around an axis introduces a distinct, continuous, and cyclic multistability (Figure 1E,F). This characteristic opens new avenues for research and application, particularly in the fields of energy absorption and intelligent structural design.

To ensure the rotational snap‐fit structure exhibits continuous multistability during rotation, several critical design conditions must be met. Specifically, the geometries of the snap‐fit and groove must be precisely aligned to ensure a seamless fit. As shown in Figure 1E, the snap‐fits and groove should exhibit identical geometry and parameter configurations, with their arrangement adhering to the principle of concentricity (centered at point *O*), and having the same insertion angle *α*, retaining angle *β*, and thickness *h*. A key design requirement is that the maximum outer diameter of the snap‐fit should match the inner diameter of the groove teeth, denoted as *r*_2_ (Figure 1E). In addition to these basic geometric relationships, the design parameters must satisfy the following mathematical conditions: the tooth angle *γ* must divide evenly into 360°, yielding a positive integer quotient *n* (i.e., 360°/*γ* = *n*); and the ratio of the number of grooves *N* to the number of snap‐fits *M* should also be a positive integer mmm (i.e., *N*/*M* = *m*). These conditions ensure the periodicity and repeatability of the structure, providing a geometric foundation for the realization of multistable deformation. Other important design parameters, such as the arc length *L* and the width *d* of the cantilever beam, are also carefully specified in Figure 1E. These structural parameters significantly impact the mechanical performance of the snap‐fit during assembly, and thus must be thoroughly considered and optimized during the design phase.

### 2.2 Parameter Analysis of Rotational Snap‐Fit Structure

Numerous factors influence the mechanical properties of snap‐fits, with the geometric parameters of the snap‐fit playing a pivotal role. Specifically, parameters such as the insertion angle *α*, the retaining angle *β*, the cross‐sectional dimensions of the cantilever beam (including width *d* and height *h*), and the length *L* of the cantilever beam, all significantly affect the mechanical properties of the snap‐fit. The impact of these parameters (e.g., insertion angle *α*, retaining angle *β*, and the cross‐sectional dimensions of the cantilever beam, including width *d* and height *h*) on the mechanical behavior of snap‐fit fasteners has been extensively investigated in prior research^[^ ^16^ ^]^ and, as such, will not be reiterated in this paper. However, given the unique symmetrical configuration of the rotational snap‐fit structure, this study will focus on the symmetry of the snap‐fit structure and the specific influence of the number of snap‐fits on its mechanical properties.

To more thoroughly analyze the mechanical behavior of the snap‐fit under torque and quantitatively examine the relationship between torque and the rotational angle, a theoretical analysis was performed in this study. Figures S5 and S6 (Supporting Information) present the mechanical model of the rotational snap‐fit under force conditions. The torque *T* can be expressed as: *T* = *Fd_F_*. According to the energy principle, the torque *T* can be derived indirectly by calculating the elastic strain energy *U* of the snap‐fit during its deformation process. This strain energy is equivalent to the work done by the external force on the system, i.e., $U=\frac{1}{2}T\psi$, where *ψ* represents the turning angle of the snap‐fit, which is a key parameter to measure the degree of rotational deformation.

In the case of the designed cantilever beam snap‐fit structure, which integrates both straight and curved beams, the total strain energy *U* can be expressed as the sum of the strain energies of the straight beam (*U*_S_) and the curved beam (*U*_C_): *U* = *U_S_* + *U_C_*. It is important to note that the strain energy of both the straight and curved beams consists of axial strain energy and bending strain energy.

By substituting specific geometric and material parameters, we get the expression of elastic strain energy *U*:

(1) $$U=\frac{1}{2}T\psi =\frac{T^{2}}{E}\left(a\frac{1}{Ar_{1}}+b\frac{r_{1}}{I}+c\frac{1}{Ar_{1}}+d\frac{r_{1}}{I}\right)$$

Simplifying this expression further, we obtain the final formula for torque *T*:

(2) T=kψ

where

(3) $$k=\frac{E}{2\left(a\frac{1}{Ar_{1}}+b\frac{r_{1}}{I}+c\frac{1}{Ar_{1}}+d\frac{r_{1}}{I}\right)}$$

**Figure 2:**
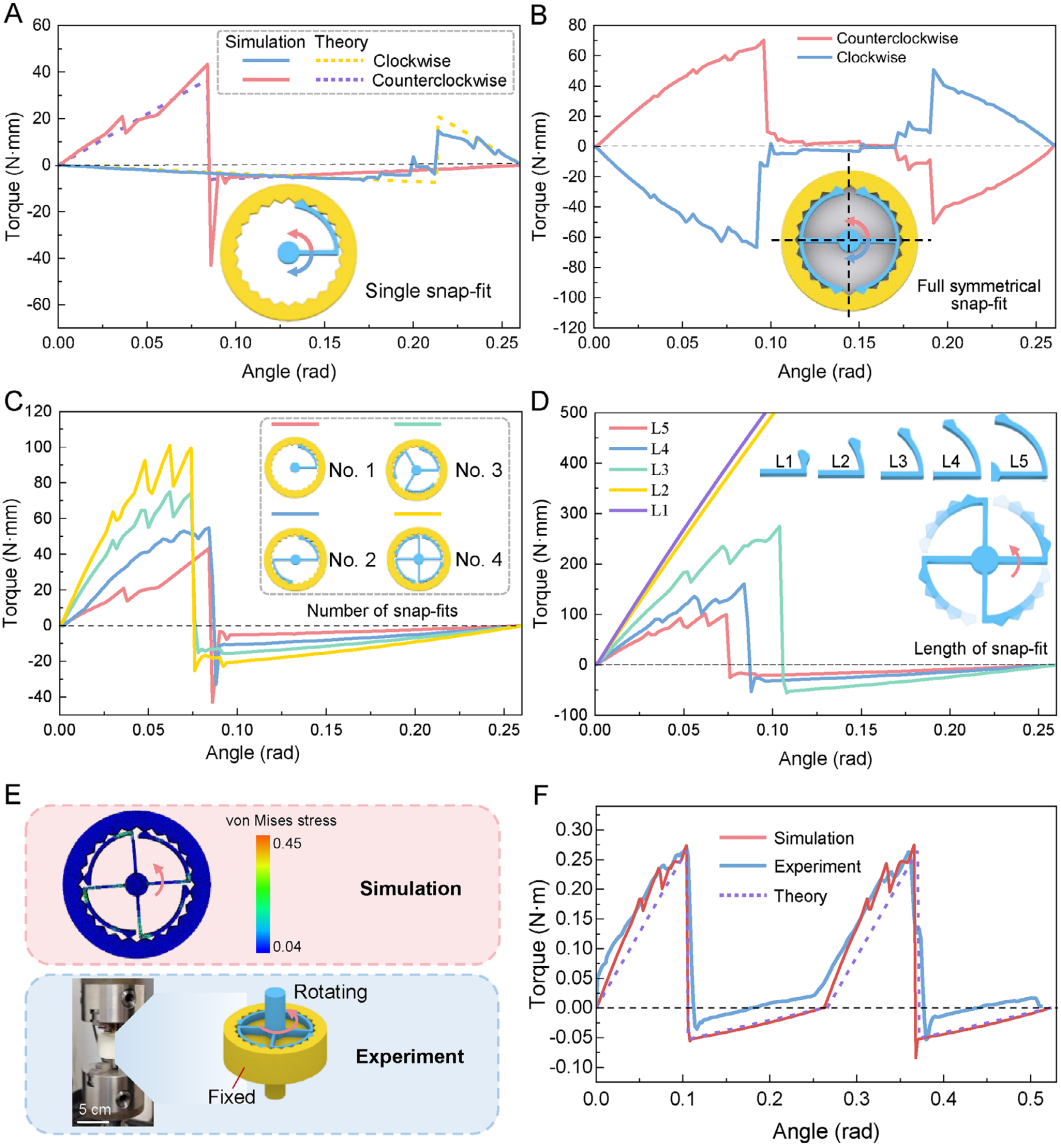
Parameter analysis of rotational snap‐fit structures. A) Individual rotational snap‐fit structure and its mechanical response characteristics (finite element simulation and theoretical analysis). The geometric parameters of the snap‐fit and groove are as follows: *α* = *β* = π/6, *θ* = 5π/12, γ = 5π/12, *r*_1_ = 17.57 mm, *r*_2_ = 20 mm, *R* = 25 mm, *L* = 25.29 mm, *d* = 1.5 mm, *h* = 4 mm. B) Symmetrically designed rotational snap‐fit structure and its mechanical response characteristics. The geometrical parameters of the snap‐fit and the groove are the same as those of A). C) Specific effects of the change in the number of rotational snap‐fits on the overall mechanical properties. The geometrical parameters of the snap‐fit and the groove are the same as those of A). D) Effects of the length of the cantilever beam on the mechanical properties of the rotational snap‐fits. Among them, *L*_1_ = 6.87 mm, *L*_2_ = 11.48 mm, *L*_3_ = 16.07 mm, *L*_4_ = 20.69 mm, *L*_5_ = 25.29 mm. The remaining geometric parameters are the same as those of A) E) Comparison pictures between the cloud diagram of stress distribution of the finite element simulation of the rotational snap‐fit and the actual torsion test. The geometrical parameters of the snap‐fit and the groove are the same as those of A) The number of snap‐fits is 4. F) Comparative analysis of mechanical characteristics of rotational snap‐fit in finite element simulation, theoretical analysis, and experiment.

The rotational stiffness *k* is a key parameter controlling the relationship between applied torque and rotational angle. This analytical formula clearly demonstrates how the parameters of the snap‐fit structure influence the system's stiffness. A thorough comparative analysis reveals that the results from simulation (details in Experimental Section) and theoretical derivation (details in Section S4, Supporting Information) exhibit a high degree of consistency (**Figure** **2**A), validating both the accuracy of the theoretical model and the reliability of the simulation results.

Figure 2A illustrates the rotational behavior of a single cantilever beam snap‐fit in a groove‐fit condition. Analysis of the torque‐angle curves reveals that the snap‐fit structure exhibits pronounced bistable behavior during torsion, both in the clockwise direction (represented by the blue curve) and the counterclockwise direction (represented by the red curve). Notably, the rotational behavior manifests a “mechanical diode” effect, wherein the maximum torque for counterclockwise rotation is substantially greater than that for clockwise rotation $\left(|T_{\max}^{Counterclockwise}\left|\gg \right|T_{\max}^{Clockwise}|\right)$. This phenomenon arises because the circular cantilever beam snap‐fit experiences higher stresses during counterclockwise rotation. Combining the force analysis of Figures S5 and S6 (Supporting Information), it can be seen that the force application points and force directions of the snap‐fit structure under counterclockwise and clockwise rotations are different, resulting in differences in the moments of the two. Through theoretical derivation, the slopes *k* of the torque and rotation angle relationships for counterclockwise and clockwise rotations satisfy *k*_1_ ≫ *k*_2_ (*k*_1_ = 437.6266, *k*_2_ = 35.1338). This means that when the same angle is rotated, the torque during counterclockwise rotation is much greater than that during clockwise rotation. Meanwhile, a comparison of the simulation results of counterclockwise/clockwise rotations has been added to the Supplementary materials, as shown in Figure S4A,B (Supporting Information). It can be clearly seen from the von Mises stress nephogram that the stress of the snap‐fit structure is greater when rotating counterclockwise.

The “mechanical diode” effect can be exploited to design ratchet mechanisms, micro‐robots, and micro‐mechanical systems. However, the structural asymmetry leads to instability, making the snap‐fit structure prone to eccentric rotation during rotation. To mitigate this issue, we propose introducing additional axial rotation constraints. We found that when the snap‐fit structure is designed with complete symmetry, the originally prominent “mechanical diode” effect becomes less obvious, as shown in Figure 2B. Specifically, in this study, four circular cantilever beam snap‐fits were arranged in a completely symmetrical layout with the *x*‐axis and *y*‐axis as the axes of symmetry. During the clockwise and counterclockwise rotation processes, the torque‐angle curves only differ in direction but are completely equal in value, that is, |*T_Counterclockwise_* |=|*T_Clockwise_* |. Thus, the rotational snap‐fit structure designed in this study serves as an effective implementation of the “mechanical diode” effect, with both symmetric and asymmetric designs offering a rich array of mechanical properties.

Additionally, this study investigates the specific influence of the number of rotational snap‐fits on the mechanical properties of the structure, with the results presented in Figure 2C. Four distinct rotational snap‐fit structures, featuring 1, 2, 3, and 4 snap‐fits, were designed for this purpose. It is noteworthy that these rotational snap‐fit structures exhibit rotational symmetry when the number of snap‐fits is 2, 3, or 4. Finite element simulations were conducted to obtain the torque‐angle curves for each configuration (Figure 2C). The results show a significant increase in the maximum torque (*T*_max_) as the number of snap‐fits increases. For the rotationally symmetric snap‐fit structures with different numbers of snap‐fits (1, 2, 3, and 4 respectively), there is a linear relationship between the torque and the number of snap‐fits, except in the region of steady‐state transition. Specifically, as shown in Figure S7 (Supporting Information), linear fitting analyses were carried out on the number of snap‐fits using the maximum torque (*T*_max_), the slope when the torque is positive, and the slope when the torque is negative, respectively (details in Section S5, Supporting Information). The determination coefficients *R* ^2^ of the fitting results are as high as 97.35%, 99.99%, and 99.99%, which strongly indicate a high degree of linear correlation between the torque and the number of snap‐fits. The emergence of this phenomenon can be attributed to the rotational symmetry of the snap‐fits and their distribution characteristics within the same plane. Owing to the rotational symmetry of the snap‐fits, each snap‐fit is capable of generating torques in the same direction when subjected to force. Meanwhile, their distribution within the same plane enables these torques to achieve linear superposition on the whole, consequently leading to an obvious linear relationship between the torque and the number of snap‐fits. However, in the actual simulation process, as the snap‐fit structures are not completely regular, this results in differences in the mesh units of each snap‐fit during meshing. Such differences render the relationship between the torque and the number of snap‐fits not strictly linear but with certain deviations. In addition, during the steady‐state transition process of the snap‐fit structure, due to the sudden change of the structure, its stress condition will become unstable. This instability makes it so that there is no obvious linear relationship between the torque and the number of snap‐fits during the steady‐state transition, and the torque value will experience large fluctuations, making it impossible to accurately describe their relationship through simple linear fitting.

Further investigation was conducted into the effect of the length of the circular cantilever beams on the rotational performance of the snap‐fit structures, with the relevant results shown in Figure 2D. Five circular cantilever beam snap‐fit structures of varying lengths (denoted *L*_1_ to *L*_5_) were designed, with *L*_1_ being the shortest and *L*_5_ the longest. The torque‐angle relationships for these structures during torsion were analyzed. It was found that due to the short arc lengths of the cantilever beams in *L*_1_ and *L*_2_, the structures generated substantial torque (over 500 N·mm) during rotation, preventing the manifestation of the multistability phenomenon. As the arc length increased (from *L*_3_ to *L*_5_), the maximum torque exhibited a decreasing trend, with *L*_5_ showing the lowest torque. This behavior can be attributed to the increased flexibility of the cantilever beams as their arc lengths increase, making longer beams more prone to deformation under the same torque. Thus, by adjusting the arc length of the cantilever beam, the torque in the rotational snap‐fit can be controlled to meet specific design requirements.

To validate the accuracy and reliability of the finite element analysis, the results from finite element simulations, theoretical calculations, and experimental tests were compared. The test samples, including the rotational snap‐fit and its corresponding groove structure, were fabricated using high‐precision stereolithography (SLA) 3D printing technology (UnionTech, China) with resin as the material. To facilitate clamping during testing, cylindrical chucks were incorporated into the design of the snap‐fit and groove, as shown in Figure 2E. Additionally, to improve the test's accuracy and prevent displacement or damage to the snap‐fits during rotation, a specialized positioning slot was designed in the groove. The rotational mechanical response (torque‐rotation curve) was tested using a Torsion 68TM‐50 rotational testing machine (INSTRON, USA). During the test, the snap‐fit and groove were securely clamped between the upper and lower chucks of the test machine (Figure 2E), with the groove structure fixed to the lower chuck and the snap‐fit structure twisted in either direction. The torque and angle data were recorded to generate the torque‐angle curve, as shown in Figure 2F. For the detailed steps and parameter settings of the test, please refer to Section 4 “Experimental Section”. A comparative analysis indicates a high degree of consistency between the experimental, theoretical, and simulation results, with only minimal deviation (Figure 2F). There are two main reasons for this minimal deviation. First, it stems from the manufacturing errors of the snap‐fit structure. During 3D printing, defects such as minor warping, shrinkage, and stair‐step patterns are inevitable, which leads to deviations in the experimental data. Secondly, it is difficult to achieve ideal testing conditions in experiments. In finite element simulations, the snap‐fit and the groove are perfectly aligned and coaxial, but it is extremely challenging to precisely achieve such perfect alignment in actual experimental tests. Despite these differences, the experimental results still show a high degree of consistency with the finite element analysis, which also indirectly confirms the reliability of the research.

### 2.3 Rotation‐Based Snap‐Fit Mechanical Metamaterials

**Figure 3:**
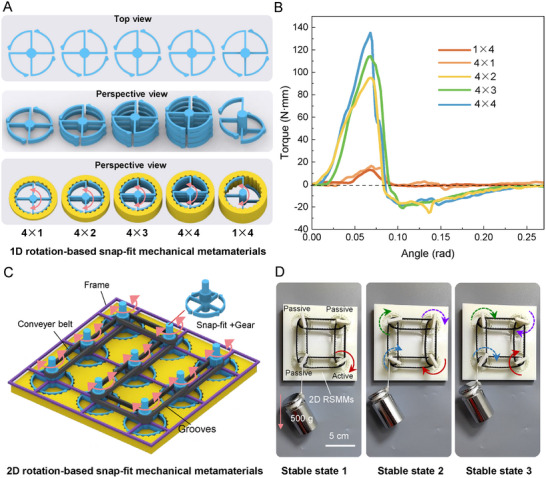
Design and mechanical response of RSMMs. A) The design scheme of 1D RSMMs is demonstrated, specifically including multiple configurations such as 4 × 1, 4 × 2, 4 × 3, 4 × 4, and 1 × 4. The geometric parameters of the snap‐fit and groove are as follows: *α* = *β* = π/6, *θ* = 5π/12, γ = 5π/12, *r*_1_ = 17.57 mm, *r*_2_ = 20 mm, *R* = 25 mm, *L* = 25.29 mm, *d* = 1.5 mm, *h* = 4 mm, *h*_4×1_ = 4 mm, *h*_4×2_ = 10 mm, *h*_4×3_ = 16 mm, *h*_4×4_ = 22 mm, *h*_1×4_ = 22 mm. B) Mechanical response properties of 1D RSMMs in different configurations. C) Design principle of 2D RSMMs: by incorporating a gear structure and utilizing a conveyor belt with a special frame with grooves, the rotational snap‐fit mechanical metamaterials were successfully constructed into 2D array structures. The geometrical parameters of the snap‐fit and the groove are the same as those of (A). D) Potential application of 2D RSMMs in the field of pattern transformation.

We constructed 1D rotation‐based snap‐fit mechanical metamaterials (RSMMs) by cascading rotational snap‐fit units. **Figure** **3**A shows the schematic structure of these RSMMs. To observe the internal structural details, the figure provides top and perspective views of the snap‐fit structure, along with a perspective view of the assembled structure (snap‐fit with the corresponding groove). We designed a series of metamaterial samples with different numbers of snap‐fits and stacked layers, using the m × n notation. Here, m represents the number of snap‐fits in a single layer, and n is the number of stacked layers. As shown in Figure 3A, we successfully designed and fabricated 1D RSMMs in 4 × 1, 4 × 2, 4 × 3, 4 × 4, and 1 × 4 configurations via 3D printing (see Experimental Section for details).

Subsequently, torque‐angle curves were obtained by assembling the snap‐fits into the corresponding grooves and testing the relationship between torque and rotational angle using a torsion tester system (Figure 3B). The analysis of the resulting curves reveals that the metamaterial system exhibits distinct rotational multistability, wherein multiple stable states emerge during the rotation of the axis. Notably, the maximum torque value (*T*_max_) increases with the number of metamaterial layers in the 1D RSMMs. This phenomenon can be attributed to the synergistic effect of the snap‐fits in each layer when subjected to force, leading to a gradual increase in the torque experienced by the central axis. Although the 4 × 1 and 1 × 4 configurations have the same total number of snap‐fits, there are slight differences in the torque‐angle response due to their different spatial layouts and errors in the manufacturing and testing processes.

Through the meticulous 1D arrangement of rotational snap‐fit structures, we achieved flexible modulation of the mechanical properties of rotation‐based snap‐fit mechanical metamaterials (RSMMs), allowing for the precise design of specific torque–angle relationships. This ability has significant potential in applications demanding precise torque control, including precision electronic switches and micromechanical systems. Specifically, by controlling the arrangement of snap‐fit structures and their interaction mechanisms, we can customize the torque‐angle response curve to meet specific needs, ensuring high control accuracy and stability in switching actions. Moreover, RSMMs possess remarkable rotational energy absorption capacity, presenting a novel way to regulate the motion of rotating objects. In applications like rotational machinery speed control, these materials can effectively absorb and dissipate rotational energy, enabling precise control of rotational speed. This characteristic is vital for improving the efficiency, stability, and responsiveness of mechanical systems, especially those requiring fine‐tuned motion control.

Building upon these results, we expanded our design scope to successfully develop 2D RSMMs (Figure 3C). To ensure effective connectivity and synergy of the snap‐fit structures in a 2D plane, we introduced a precision‐designed gear structure and employed a conveyor belt mechanism, along with a frame system equipped with grooves, to organize the units into a structured 2D array. This design not only ensures the stable connection of the snap‐fit structure in a 2D space but also enhances the structure's deformation ability among various shapes. The 2D RSMMs were meticulously fabricated using advanced 3D printing techniques, with their physical manifestation shown in Figure 3D and Movie S3 (Supporting Information). To assess their performance, a 500 g weight was suspended from the passive drive unit (Figure 3D). The experimental results demonstrate that the passive unit rotates synchronously with the active unit, exhibiting excellent coordination and stability. Notably, we successfully demonstrated three stable states of the 2D RSMMs under a 500 g load, underscoring the system's ability to maintain its stable configuration even under substantial external forces, a feat difficult to achieve with conventional conveyor mechanisms. This validates the unique mechanical advantages and structural stability of RSMMs.

Metamaterials manifest unique capabilities in visual display and information transfer realms, functioning analogously to dynamic displays. More precisely, by affixing desired display components onto the top of the snap‐fit structure and governing the twist angles of the snap‐fits, the top structure can be maneuvered to various angles and positions, thus engendering rich and diverse visual effects. This characteristic begets novel creative openings for visual arts, dynamic advertising design, and information visualization. Moreover, 2D RSMMs possess extensive potential applications in hypersurfaces and topological research. By integrating particular units into the metamaterial structure and rotating them at different angles, a multiplicity of surface morphologies and topologies can be generated, furnishing new perspectives and experimental platforms for material science, nanotechnology, and photonics. Significantly, metamaterials can also be harnessed for information encryption and concealment. By predefining distinct angle sequences, specific information or patterns can be exhibited, facilitating the secure and confidential transmission and display of information. This trait holds great promise in fields such as information security, anti‐counterfeiting technologies, and communication.

### 2.4 Multistable Meta‐Lines and Multistable Shape Deformations

**Figure 4:**
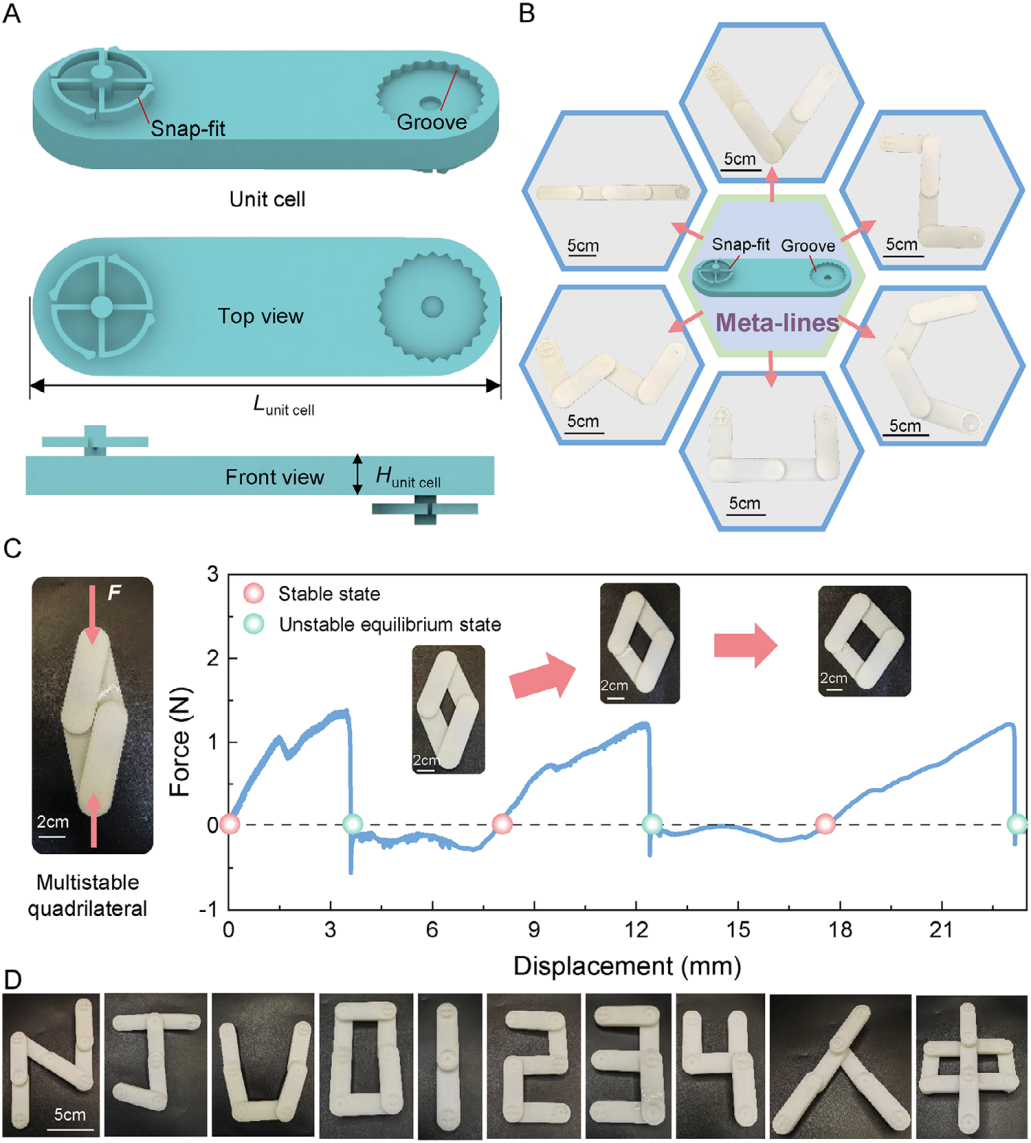
Multistable meta‐lines and multistable shape deformation. A) A unit cell consisting of a rotational multistable snap‐fit with a corresponding groove structure. The geometric parameters of the unit cell are as follows: *α* = *β* = π/6, *θ* = 5π/12, γ = 5π/12, *r*_1_ = 8.79 mm, *r*_2_ = 10 mm, *R* = 12.5 mm, *L* = 12.65 mm, *d* = 0.75 mm, *h* = 2 mm, *L*_unit cell_ = 85 mm, *H*_unit cell_ = 8 mm. B) The design concept of the meta‐lines and the mechanism of the shape change it induces is shown. C) Mechanical response of a multistable quadrilateral during steady state transition. D) The exhibit is able to construct diverse shapes including but not limited to letters, numbers, and Chinese character patterns by combining multiple unit cells.

For a more illustrative exposition of the shape deformation traits of rotational multistable structures, we devised the rotational multistable snap‐fit and groove structures in the form of unit cells. The configuration of the rotational multistable unit cell is depicted in **Figure** **4**A. To facilitate the observation of the internal structural details, a top and front view of the cell unit is also provided, with two sets of rotating multistable snap‐fits and groove structures symmetrically distributed on both sides of the cell unit. This deliberate arrangement serves dual purposes: it streamlines the assembly procedure and simultaneously bolsters the structural stability and equilibrium when subjected to external forces. Consequently, it paves the way for the attainment of intricate geometric transformations.

This design facilitates the efficient assembly of various snap‐fit chains through a torsional mechanism, wherein the torsional flexibility between the snap‐fit and groove provides hinge‐like rotational capabilities to the chain nodes. These nodes exhibit multiple homeostatic properties, meaning they can stabilize and maintain specific configurations. When multiple snap‐fit chains are assembled in unison, they can exhibit a diverse range of morphological features (Figure 4B). All samples were fabricated by 3D printing technology (details in the Experimental Section). By employing a carefully designed assembly strategy, four independent unit cells can form various stable configurations that are interconvertible. As demonstrated in Figure 4B, these stable states include but are not limited to, a straight line, a V‐shape, a zigzag, a C‐shape, a concave shape, and a W‐shape. We collectively refer to this series of highly morphologically variable snap‐fit chains as “meta‐lines,” which not only showcase cross‐dimensional shape deformation capabilities but also expand the applicability of torsional snap‐fit structures by enabling flexible transitions from 1D linear structures to 2D planar and even 3D forms.

To more characterize the mechanical properties of the meta‐lines during the transition to multistable configurations, we designed a multistable geometric configuration (a quadrilateral structure) assembled from four independent unit cells and conducted extensive mechanical testing using a universal testing machine to quantify its response during the shape evolution stage. In the experimental setup, one vertex of the quadrilateral was fixed at the lower fixture of the testing machine, while the opposite vertex, located on the diagonal, was clamped by the upper fixture, to which a predetermined downward displacement was applied (left side of Figure 4C). By recording and analyzing the force‐displacement curves during the loading process (right side of Figure 4C), we observed that the angle of the clamped vertex increased progressively as upward displacement was applied, with the entire structure undergoing three stable configurational transitions in sequence. The red‐marked points on the curves on the right side of Figure 4C denote the stabilization points of these configurations, marking the achievement of mechanical equilibrium as the structure transitions between different conformations.

To further validate the concept of meta‐lines, we fabricated multiple snap‐fit chain units using 3D printing technology and successfully constructed various shapes by carefully assembling and adjusting the snap torsion angle (shown in Figure 4D). These shapes include not only numerical figures (e.g., 0, 1, 2, …, 9), letters (e.g., N, J, U), but also simulations of Chinese character structures (e.g., “ren (人)”, “zhong (中)”). These results conclusively demonstrate the significant advantages and broad application potential of meta‐lines in the field of graphic reconstruction.

Meta‐lines represent a novel category of deformable materials capable of achieving complex morphological changes through a simple twisting mechanism. The design and fabrication of such materials open up new possibilities for future applications in fields such as soft robotics, deformable structures, and smart textiles.

### 2.5 Applications of RSMMs

The rotational snap‐fit structure and RSMMs exhibit a wide range of potential applications due to their distinctive mechanical behavior. These materials enable efficient energy absorption and are particularly valuable in robotics, especially in the design and functional implementation of robotic grippers.

**Figure 5:**
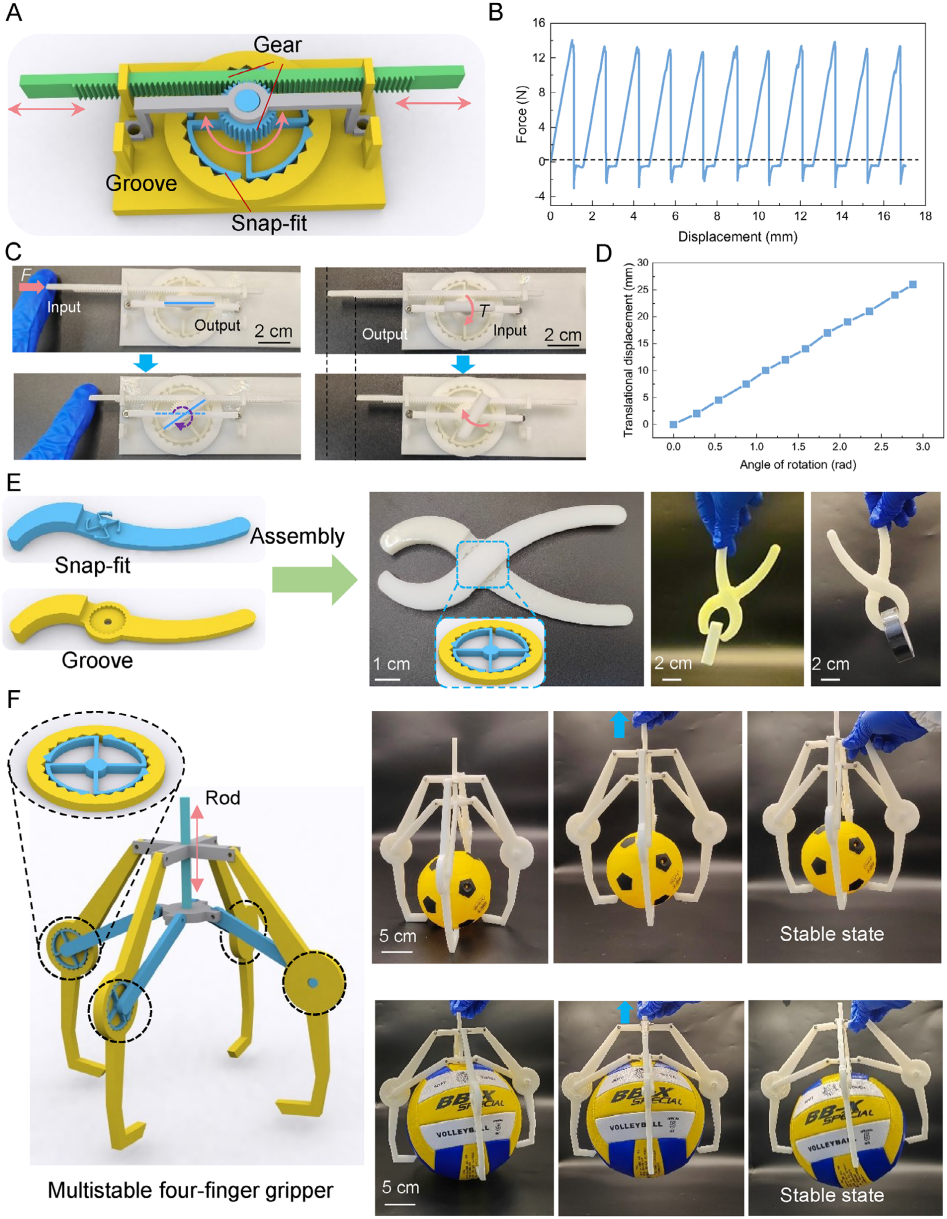
Application of RSMMs. A) An effective conversion mechanism between torsional multistability and translational multistability is achieved through precision‐designed gear mechanisms. The geometric parameters of the snap‐fit and groove are as follows: *α* = *β* = π/6, *θ* = 5π/12, γ = 5π/12, *r*_1_ = 17.57 mm, *r*_2_ = 20 mm, *R* = 25 mm, *L* = 25.29 mm, *d* = 1.5 mm, *h* = 4 mm. The number of snap‐fits is 4. B) The mechanical response characteristics during the transition from torsional multistability to translational multistability. C) Conversion between translational and rotational motion of the mechanism: (Left) The mechanism takes translational motion as the input and rotational motion as the output. (Right) The mechanism takes rotational motion as the input and translational motion as the output. D) Relationship between rotational angle and translational displacement. E) A two‐finger grasping hand built based on the torsional multistability principle, and a demonstration example of grasping operation (eraser and tape). F) A four‐finger grasping hand design based on torsional multistability, and its application demonstration in actual grasping tasks (soccer ball and basketball).

The rotational snap‐fit structure, designed based on a rotational input mechanism, is characterized by its unique rotational multistable behavior. By integrating this structure with a spur gear system, we successfully developed a mechanism capable of transitioning between rotational and linear motion, as illustrated in **Figure** **5**A. Notably, during this complex transition process, the mechanism maintains configuration flexibility and multistability through its engineered structural design, ensuring stable and reliable system operation. To further evaluate the mechanism's performance in mechanical environments, it was installed on a precision universal testing machine, as shown in Figure S2B and Movie S4 (Supporting Information). Systematic mechanical response tests under external loads were conducted, and the resulting force‐displacement curves are presented in Figure 5B. The curves clearly demonstrate that the mechanism exhibits pronounced multistable characteristics during loading, confirming its structural stability and conversion efficiency. In particular, this conversion mechanism not only has the absorption capacity of rotational energy but also can realize the absorption of translational energy.

To achieve a more intuitive and accurate presentation of the conversion mechanism between translational and rotational motions, the mechanism shown in Figure 5A was taken as the research object. The translational and rotational motions were respectively set as the input modes to observe the corresponding changes at the output end, and the results are shown in Figure 5C. Figure 5C (left) illustrates the situation where the spur gear in the mechanism is used as the input end and the horizontal beam on the snap‐fit is used as the output end. When the spur gear at the input end is in a translational state, the horizontal beam at the output end correspondingly rotates. In contrast, in Figure 5C (right), the input and output ends are reversed, that is, the horizontal beam on the latch is used as the input end and the spur gear is used as the output end. At this time, by rotating the horizontal beam at the input end, the translational phenomenon of the spur gear at the output end can be clearly observed. Meanwhile, the relationship between the rotation angle and the translational displacement was recorded, and the result is shown in Figure 5D. It can be found that a linear relationship exists between them. The reason is that during the meshing process of the spur gear and the spur wheel, the spur gear is tangent to the spur wheel and moves horizontally along the tangent point of the spur wheel. That is, for every certain angle the spur wheel rotates, the spur gear moves a fixed distance. It can be seen that this mechanism can effectively achieve a high‐efficiency conversion between the rotational mode and the translational mode, providing a practical and feasible way for mechanical motion conversion in related fields. The integration of the rotational snap‐fit structure with the spur gear system expands the application potential of multistable structures and provides significant theoretical and practical support for advancements in energy management technologies.

Furthermore, this study introduces the multistable rotational snap‐fit structure into robotic gripper design by incorporating it into the robot's kinematic joints and connecting it via a linkage mechanism. As depicted in Figure 5E,F and Movie S5 (Supporting Information), two‐finger and three‐finger gripper configurations were meticulously designed. The incorporation of the multistable snap‐fit structure allows the gripper system to maintain a stable grasp without requiring continuous external energy supply after successfully capturing an object. This feature significantly minimizes the risk of damage to delicate objects due to excessive gripping force and effectively reduces energy consumption, offering a substantial advantage in terms of energy efficiency.

Figure 5E,F and Movie S5 (Supporting Information) showcase the performance of the designed and fabricated gripping system in practical applications, demonstrating stable gripping of eraser, tape, soccer ball and basketball. These results confirm the design's effectiveness and practicality. Overall, the experimental outcomes validate the potential applications of rotational multistable mechanical metamaterials in robotic grippers, highlighting their advantages in improving operational efficiency and reducing energy consumption. All samples were fabricated by 3D printing technology (details in the Experimental Section).

## 3 Conclusion

In this study, based on the straight‐beam snap‐fit, a rotational snap‐fit structure featuring a circular cantilever beam snap‐fit and a circular groove is designed, breaking through traditional limitations. This structure demonstrates multistability during torsion. The torque‐angle relationship similar to the force‐displacement relationship of the straight‐beam snap‐fit, and it can rotate continuously around the axis, generating continuous cyclic multistability. This offers new prospects for energy absorption and intelligent structural design. To achieve multistability deformation, the design must ensure precise matching of the geometric features of the snap‐fit and groove, meeting a series of key conditions such as concentric circle arrangement, consistent dimensions, and specific mathematical relationships. Structural parameters have a substantial impact on mechanical properties and should be fully considered in the design.

The mechanical properties of the rotational snap‐fit structure are notably affected by geometric parameters. It shows an interesting “mechanical diode” effect during rotation. Theoretical derivation reveals a quantitative relationship between torque and rotation angle, which highly agrees with simulation results. Increasing the number of snaps significantly raises the maximum torque, and rotational symmetry leads to linear torque superposition. The arc length of the cantilever beam influences the torque: short arc lengths generate high torque, while long arc lengths reduce torque due to increased flexibility. Experiments verify the accuracy of finite element analysis and theoretical calculation results, validating the reliability of the analysis method.

The rotational snap‐fit units were assembled to fabricate 1D RSMMs. Samples with diverse configurations were meticulously designed and fabricated. The 1D RSMMs possess multistable properties, wherein the maximum torque value escalates with the increment in the number of layers. Such characteristics render them suitable for applications in precision electronic switches and micromechanical systems. For 2D RSMMs, a precision gear and conveyor belt mechanism was employed to enable the cooperative operation of snap‐fits within the 2D plane. This mechanism endows them with synchronization and stability, allowing them to maintain a multistable state under load. These properties make 2D RSMMs apt for visual display, information transmission, metasurface, and topological research, as well as information security applications.

To demonstrate the deformation characteristics of RSMMs, snap‐fit, and groove structures were designed as unit cells. This design strategy optimized the assembly process, guaranteed force stability, and facilitated the attainment of complex geometric transformations. Multiple unit cells coalesced to form meta‐lines, which showcased cross‐dimensional shape deformation capabilities. Mechanical tests demonstrated that a quadrilateral structure underwent three stable configurations during the loading process. Additionally, 3D printing technology validated the superiority of meta‐lines in the field of graphic reconstruction, enabling the construction of numbers, letters, and Chinese characters.

RSMMs exhibit potential in energy absorption and robotics, attributable to their distinctive mechanical behavior. The rotational snap‐fit structures, characterized by unique rotational multistability, when integrated with spur gears, can effectuate efficient conversion from rotational to linear motion. This integration not only preserves configuration flexibility but also allows for the testing and validation of stability and conversion efficiency, and further facilitates efficient energy capture. When incorporated into the robot gripping mechanism, deployed at joints, and interconnected via connecting rods, RSMMs enable stable gripping, conserve energy, and prove effective in handling multiple object types, thus accentuating their application advantages.

Looking to the future, RSMMs are poised to pioneer more innovative applications. In robotics, optimized RSMMs are anticipated to enhance the intelligence and flexibility of gripping mechanisms, enabling them to adapt to increasingly complex environments. In the domain of energy management and conversion, their efficient energy utilization properties hold the promise of revolutionizing energy storage, vibration damping, and power transmission systems. Concurrently, their cross‐dimensional deformation capabilities will spawn innovation opportunities in areas like soft robots and smart textiles. Overall, research on this material and technology will fuel scientific and technological advancement, contribute to sustainable development and the intelligent transformation of industries, and beckon more innovative applications and breakthroughs in the future.

## 4 Experimental Section

### Fabrication

The samples prepared for this experiment were printed using stereolithography (SLA) 3D printing technology. The equipment used was a Lite 800 industrial 3D printer (Shanghai UnionTech Technology Co., Ltd., Shanghai, China) (Figure S1, Supporting Information). The material used was Somos Imagine 8000 photosensitive resin. Its Young's Modulus was 2510 MPa and Poisson's Ratio is 0.41 (detailed material property parameters are provided in Supplementary Materials Table S1, Supporting Information). 2D rotational snap‐fit mechanical metamaterials were constructed by meshing the synchronized toothed belt with the gears on the snap‐fit to realize the connection of the snap‐fit structure in 2D space.

### Testing

A commercial torsion testing machine (INSTRON 68TM‐50, USA) was used to obtain torque‐angle characteristic curves of the snap‐fit structure during rotation (Figure S2A and Movie S1, Supporting Information). In the experiment, a chuck fixture was used to fix the snap‐fit and groove assemblies on the testing machine, and three rotations each in clockwise and counterclockwise directions were performed at a constant rotational speed of 0.2 rad s^−1^, and the angle and torque data were recorded at the same time.

In addition, in order to investigate the translational mechanical properties of the samples, a commercial universal testing machine manufactured by Shimadzu Corporation (Japan) was used to conduct static uniaxial tensile experiments, and the force‐displacement curves were obtained by measuring and recording the displacement and load data at a displacement rate of 10 mm min^−1^ (Figure S2B and Movie S4, Supporting Information).

In order to visually observe the process of grasping objects by the gripper, relevant videos were captured with a normal camera at a resolution of 1080p and a speed of 30 frames per second.

### FEM

In this study, a series of finite element simulations (FEAs) were executed using the commercial finite element analysis software ABAQUS (SIMULIA) (Figure S3, Supporting Information). A variety of snap‐fit models with different geometrical parameters were constructed and their deformation behavior and torque‐angle response under quasi‐static conditions were analyzed in depth. The simulation results are presented in the form of post‐deformation stress clouds (Figure S3D and Movie S2, Supporting Information). The energy absorption values are obtained by calculating the integral area of the region enclosed by the torque‐turn angle curve and the x‐axis.

## Conflict of Interest

The authors declare no conflict of interest.

## Author Contributions

R.X. performed methodology, formal analysis, investigation, and wrote the original draft; Y.H. performed methodology and wrote the original draft; C.C. performed methodology and wrote the original draft; J.S. performed methodology and wrote the original draft; X.L. performed conceptualization, investigation, supervision, and wrote and reviewed the original draft and edited the manuscript; M.‐H.L.; acquired resources, performed validation, wrote and reviewed the draft and edited the manuscript; Y.‐F.C. acquired resources, wrote and reviewed the draft, and edited the manuscript.

## Supporting information



Supporting Information

Supplemental Movie 1

Supplemental Movie 2

Supplemental Movie 3

Supplemental Movie 4

Supplemental Movie 5

Supporting Information

## Data Availability

The data that support the findings of this study are available from the corresponding author upon reasonable request.
