# Supplementary material for: Rotation‐Based Snap‐Fit Mechanical Metamaterials
Source: Adv Sci (Weinh). 2025 Mar 24;12(19):2501749. doi: 10.1002/advs.202501749 (PMC12097127; doi:10.1002/advs.202501749)
Supplement: Supplementary file 1 — Supporting Information [file ADVS-12-2501749-s001.docx]

# Supporting Information for

# Rotation-based Snap-fit Mechanical Metamaterials

Rui Xu^1^, Yulong He^1^, Chuanqing Chen^1^, Jiapeng Sun^1^, Xin Li^2,*^, Ming-Hui Lu^1,*^, Yan-Feng Chen^1^

1. College of Engineering and Applied Sciences, Nanjing University, Nanjing, Jiangsu 210093, PR China

2. School of Mechanical Engineering, Nanjing University of Science and Technology, Nanjing, 210094, China

*Corresponding authors, E-mail: xinli@njust.edu.cn, luminghui@nju.edu.cn

**This file includes:**

**Supplementary sections**

[Section S1 Specimen preparation process 3](#_Toc190790265)

[Section S2 Mechanical property test 5](#_Toc190790266)

[Section S3 Finite Element Simulation Process 7](#_Toc190790267)

[Section S4 Theoretical analysis 9](#_Toc190790268)

[Section S5 Relationship between the number of snap-fits and torque 13](#_Toc190790269)

**Figure S1 to S5**

**Table S1**

**Captions for Supplementary Movies S1 to S5**

## Section S1 Specimen preparation process

First, 3D CAD models of the part were accurately constructed (**Figure S1A**), and these models were subsequently converted into an STL file, which uses a triangular mesh to meticulously depict the external contours of the part, and is a standard file format in the 3D printing field. Using specialized 3D printing pre-processing software, these models were further converted into a layered file with support structures cleverly added to enhance print stability. Once the layering is complete, all key files are loaded into the device computer and uploaded to the virtual build platform through the process software for precise positioning.

Before the print job starts, the squeegee is thoroughly cleaned of cured resin residue to ensure print quality. The part manufacturing process is highly automated and can be run efficiently with little or no human intervention. At the same time, a sufficient amount of resin is required to maintain stable operation of the equipment. In addition, the temperature (maintained at 22-26 ℃) and humidity (below 40%) of the processing environment are strictly controlled to optimize the printing results.

In this study, a lite 800 stereolithography industrial 3D printer (Shanghai UnionTech Technology Co., Ltd., Shanghai, China) was used for sample preparation. As shown in **Figure S1B**, the UV laser was precisely scanned on the surface of liquid photosensitive resin (Somos® Imagine 8000) to trigger the photopolymerization reaction of the resin thin layer to form a solid thin layer cross-section. The complete 3D models (i.e., sample) are ultimately constructed by curing and overlaying the layers one by one.

After printing, the part is carefully removed and the support structure and uncured resin adhering to the surface are removed by cleaning, using alcohol as the cleaning medium. To further strengthen the part, the cleaned part is cured for 20 minutes in a UV light curing chamber. Finally, the surface of the part was finely sanded to obtain a smooth and flat final test sample (**Figure S1C**). Table S1 details the key performance parameters of the light-curing resins used, including density, Young’s modulus, and Poisson’s ratio, which provide an important basis for the physical property analysis of the samples.


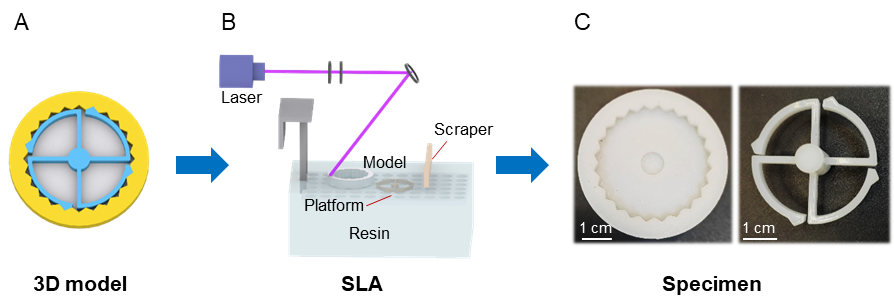


**Figure S1** **The** **process of specimen preparation using stereolithography (SLA) 3D printing technology.** (A) 3D models of the desired part are constructed in computer-aided design (CAD) software. (B) The 3D model is processed before printing, including slicing, adding support structures, etc., and the processed model is imported into the SLA 3D printer. The printer processes the model layer by layer according to preset parameters until the entire printing process is completed. (C) After printing, post-processing is performed, including removing the support structure, cleaning the surface residue, and curing as necessary, etc., to finally obtain a sample that meets the requirements.

**Table S1** The performance parameters of the materials.

|  | Photosensitive Resin |
| --- | --- |
| Density (g/cm^3^) | 1.16 |
| Young’s Modulus (MPa) | 2510 |
| Poisson’s Ratio | 0.41 |

## Section S2 Mechanical property test

In order to evaluate the mechanical behavior of the rotational snap-fit structure, a commercial torsion test equipment (INSTRON 68TM-50, USA) was used in this study with the aim of obtaining the torque-angle characteristic curve of the structure under rotational action. As shown in **Figure S2A and Movie S1**, the following measures were taken to ensure the robustness of the test and the reliability of the data: first, three-jaw chucks were utilized to implement the clamping to enhance the clamping effect; second, cylindrical fixtures were added to the test specimen, and the locating holes were designed to circumvent the eccentric rotation phenomenon. The experimental steps include: mounting the snap-fit and groove assemblies firmly on the testing machine by means of three-jaw chucks, precisely aligning the center of the snap-fit using the locating holes, and applying lubricant inside the holes to reduce frictional resistance. Subsequently, three rotations in clockwise and counterclockwise directions were carried out at a constant angular velocity of 0.2 rad/s, and the rotational angle and torque data were recorded accurately at the same time.

In order to further investigate the mechanical properties of the rotational snap-fit structure when converted to translation by spur gear, a commercial universal testing machine (Shimadzu, Japan) was used in this study to perform static uniaxial tensile testing, as shown in **Figure S2B and Movie S4**. To ensure the uniaxial displacement of the samples during the stretching process, fixtures were added at each end of the spur gear and the bay and calibrated to the same height and horizontal position. During the experiment, the load was applied at a constant displacement rate of 10 mm/min, and the displacement and load data were recorded accurately at the same time, from which the force-displacement response curves under uniaxial tensile conditions were plotted.


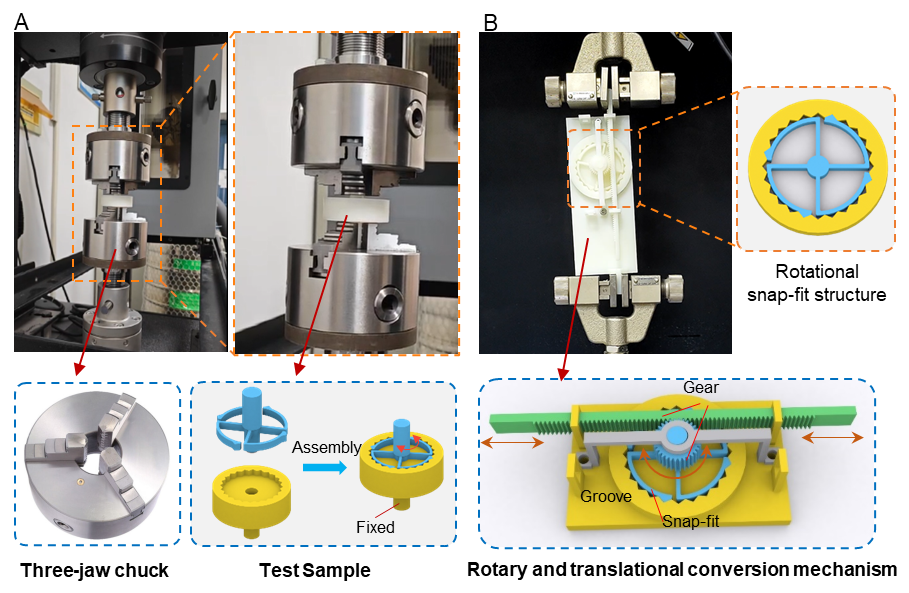


**Figure S2** **Mechanical properties testing procedure of the samples.** (A) Torsional performance evaluation: a torsion testing machine was utilized with the aim of obtaining and analyzing the torque-rotation angle characteristic curve of the samples during rotation. (B) Tensile property analysis: a universal testing machine was utilized in order to explore and record the force-displacement characteristic curves of the samples under uniaxial tensile conditions.

## Section S3 Finite Element Simulation Process

In this study, we used the commercial software ABAQUS (SIMULIA) for finite element analysis (FEA) to investigate the mechanical behavior of the snap-fit structure during torsion. **Figure S3** shows the process of this FEA simulation in detail. First, we constructed several snap-fit models with different geometrical features (**Figure S2A**) and analyzed their deformation characteristics as well as torque-angle relationships under quasi-static conditions. During the model construction stage, we implemented chamfering at the contact edges to ensure the stability and convergence of the simulation process. The material properties were set as Young’s modulus 2510 MPa, Poisson’s ratio 0.41, and density 1.16×10^-9 t/mm³. For the solution, we chose the ABAQUS/Standard (Dynamic, Explicit) solver and considered geometric nonlinear effects. In defining the interaction between the structures, we set the contact properties through the interaction module. The tangential contact follows a penalized friction formulation with a friction coefficient set to 0.05, while the normal contact is modeled as a “Hard” Contact and specified as General contact (Explicit). For rotational loading, a reference point (RP) is defined at the center of rotation of the snap-fit structure and connected to the loading surface by surface coupling technique. The boundary conditions were set as V1=V2=V3=VR1=VR2=0, while the rotational angular velocity VR3 was set to 0.2 rad/s (**Figure S2B**). For meshing, we used an eight-node linear hexahedral cell (C3D8R) to discretize the snap-fit model (**Figure S2C**), and ensured the accuracy and convergence of the computational results by refining the meshing strategy (seed Part of 0.5 and seed edge of 0.1). After completing the finite element simulation, we obtained a cloud view of the stress distribution after deformation (**Figure S2D and Movie S2**) and recorded the correspondence between the torque and the turning angle in each simulation. In addition, by calculating the integral area enclosed by the force-displacement curve and the *x*-axis, we further derived the energy absorption *E*. In order to compare the difference between counterclockwise rotation and clockwise rotation of the snap-fit structure, **Figure S4A-B** also shows the simulated von Mises stress distribution cloud. Through these detailed simulation steps, we were able to accurately simulate and analyze the mechanical behaviors of the snap structure during the rotation process, which provided a scientific basis for the design optimization.


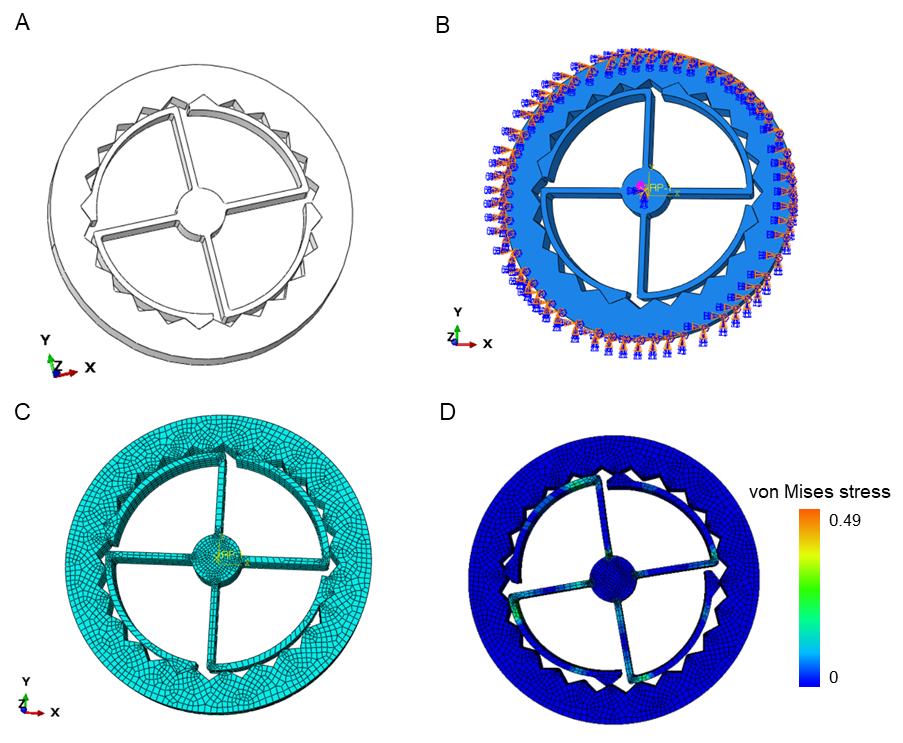


**Figure S3** **Finite element simulation process for the torsional motion of the snap-fit structure.** (A) Model construction and material property assignment. (B) Setting of boundary conditions. (C) Meshing. (D) Simulation results presentation: stress distribution after deformation.


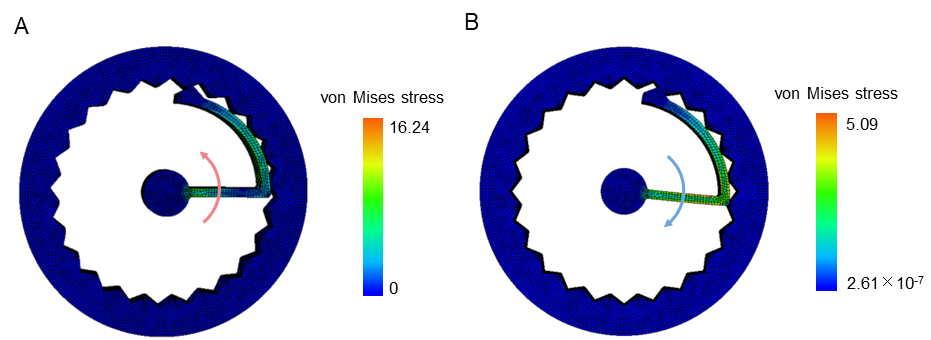


**Figure S4** von Mises stress distribution of single snap-fit structure during rotation: (A) counterclockwise; (B) Clockwise.

## Section S4 Theoretical analysis

The mechanical response of curved cantilever beams under torque is theoretically analyzed, aiming to clarify the intrinsic connection between the torque and the torsional angle. In view of the fact that the deformation produced by the cantilever beam snap-fit during rotation is much less than its original size, the small deformation assumption is used here, and the initial configuration is the configuration for analysis.


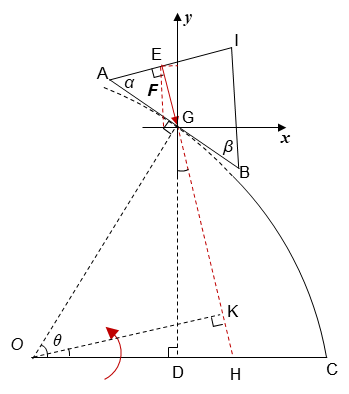


**Figure S5** Force analysis of the insertion surface of the snap-fit in contact with the groove when the rotational snap-fit is turned counterclockwise.

**Figure S5** visualizes the force state at the contact interface between the insertion surface of snap-fit and the groove when the torsion snap-fit is rotated counterclockwise. At this time, ***F*** represents the total force acting on the twisted snap, while ***F****_x_* and ***F****_y_* are the components of this combined force in the *x*-axis and *y*-axis directions, respectively. Based on the geometric relationship in **Figure S5**, the following angular expression can be derived:

∠DGH=α+θ-π/2 (1)

∠GOK=π/2-α (2)

Therefore, the relation between ***F****_x_* and ***F****_x_* can be obtained:

$F_{x}=F\sin(\theta+\alpha-\pi/2)$ (3)

$F_{y}=F\cos(\theta+\alpha-\pi/2)$ (4)

Based on the above angular relationship, we further derive an expression for the torque *T*:

$T=Fd_{F}=Fr_{1}\cos(\frac{\pi}{2}-\alpha)$ (5)

With a small increment in the torsional angle, the work of torque T is transferred into the elastic energy of the snap-fit:

$Td\psi=dU$ (6)

i.e.

$U=\frac{1}{2}T\psi$ (7)

where *U* represents the elastic strain energy stored in the torsion snap-fit during deformation, which is equal to the work done by the external force. *ψ* is the turning angle of the snap-fit.

Given that the designed cantilever beam snap-fit structure incorporates both straight and curved beams, the elastic strain energy *U* can be further subdivided into the sum of the straight beam (OC) strain energy *U*_S_ and the curved beam (GC) strain energy *U*_C_, i.e.:

$U=U_{S}+U_{C}$ (8)

Among them, the straight beam strain energy *U*_S_ covers both axial strain energy and bending strain energy, which is expressed as:

$U_{S}=\frac{F_{x}^{2}r_{1}}{2EA}+\int_{0}^{r_{1}} \frac{[F_{x}r_{1}\sin\theta+F_{y}(r_{1}\cos\theta-x)]^{2}dx}{2EI}$ (9)

Similarly, the curved beam strain energy *U*_C_ also contains both axial and bending strain energy, i.e.:

 (10)

Where

 (11)

In summary, the final expression for the elastic strain energy *U* can be derived as:

 (12)

where the parameters are taken as follows: *α* = *β* = π/6; *θ* = 5π/12; *r*_1_ = 17.82mm; *h* = 4mm; *d* = 1.5mm; Young’s modulus *E* = 2510 MPa, cross-sectional area *A* = 6 mm², and cross-sectional moment of inertia *I* = 1.125 mm⁴.

These parameters were calculated by substituting them into Eq. (12):

$U=\frac{1}{2}T\psi=\frac{T^{2}}{E}(a_{1}\frac{1}{Ar_{1}}+b_{1}\frac{r_{1}}{I}+c_{1}\frac{1}{Ar_{1}}\text{+}d_{1}\frac{r_{1}}{I})$ (13)

Therefore, the expression for the torque *T* is:

$T=k_{1}\psi$ (14)

Where

$k_{1}=\frac{E}{2\left(a_{1}\frac{1}{Ar_{1}}+b_{1}\frac{r_{1}}{I}+c_{1}\frac{1}{Ar_{1}}\text{+}d_{1}\frac{r_{1}}{I}\right)}$ (15)

Bringing in the specific parameters gives: *a*_1_ = 0.1340; *b*_1_ = 0.1561; *c*_1_ = 1.9920; *d*_1_ = 0.2423; *k*_1_ = 437.6266.


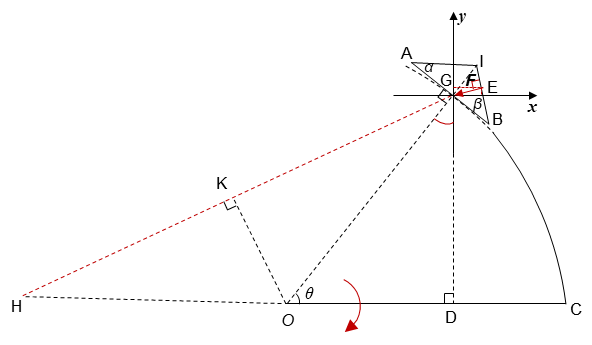


**Figure S6** Force analysis of the retaining surface of the snap-fit in contact with the groove when the rotational snap-fit is turned clockwise.

For the clockwise rotation of the snap-fit, the force state of the contact interface between the insertion surface of snap-fit and the groove is shown in **Figure S6**. Based on the geometric relationship in **Figure S6**, the following angular expression can be derived:

∠DGH= β-θ+π/2 (16)

∠OGH=β (17)

Therefore, the relation between ***F****_x_* and ***F****_y_* can be obtained:

$F_{x}=F\sin(\beta-\theta+\pi/2)$ (18)

$F_{y}=F\cos(\beta-\theta+\pi/2)$ (19)

Based on the above angular relationship, we further derive the expression for the torque *T*. The expression for the torque *T* is given by

$Fd_{F}=Fr_{1}\sin\beta=T$ (20)

Here, the derivation process of the elastic strain energy of the cantilever beam is consistent with the above, and the difference lies in the difference of the bending moment *M*_θ_ when calculating the bending strain energy of the curved beam, and the expression of *M*_θ_ is:

 (21)

Therefore, the expression for the torque *T* is calculated by bringing in the parameters as:

$T=k_{2}\psi$ (22)

Where

$k_{2}=\frac{E}{2\left(a_{2}\frac{1}{Ar_{1}}+b_{2}\frac{r_{1}}{I}+c_{2}\frac{1}{Ar_{1}}\text{+}d_{2}\frac{r_{1}}{I}\right)}$ (23)

Substitute the specific parameters gives: *a*_2_ = 0.1340; *b*_2_ = 0.6086; *c*_2_ = 2.2420; *d*_2_ = 1.6451; into Eq. (23). The *k*_2_ = 35.1338. The torsional stiffness of the snap-fit *k*_2_ is the most important parameter, which controls the relation between torque and torsional anger.

The above analysis gives an analytical relation between the torsional stiffness of the snap-fit and its structures parameters, which provides guidance for the subsequent experimental design and structural optimization.

## Section S5 Relationship between the number of snap-fits and torque

**
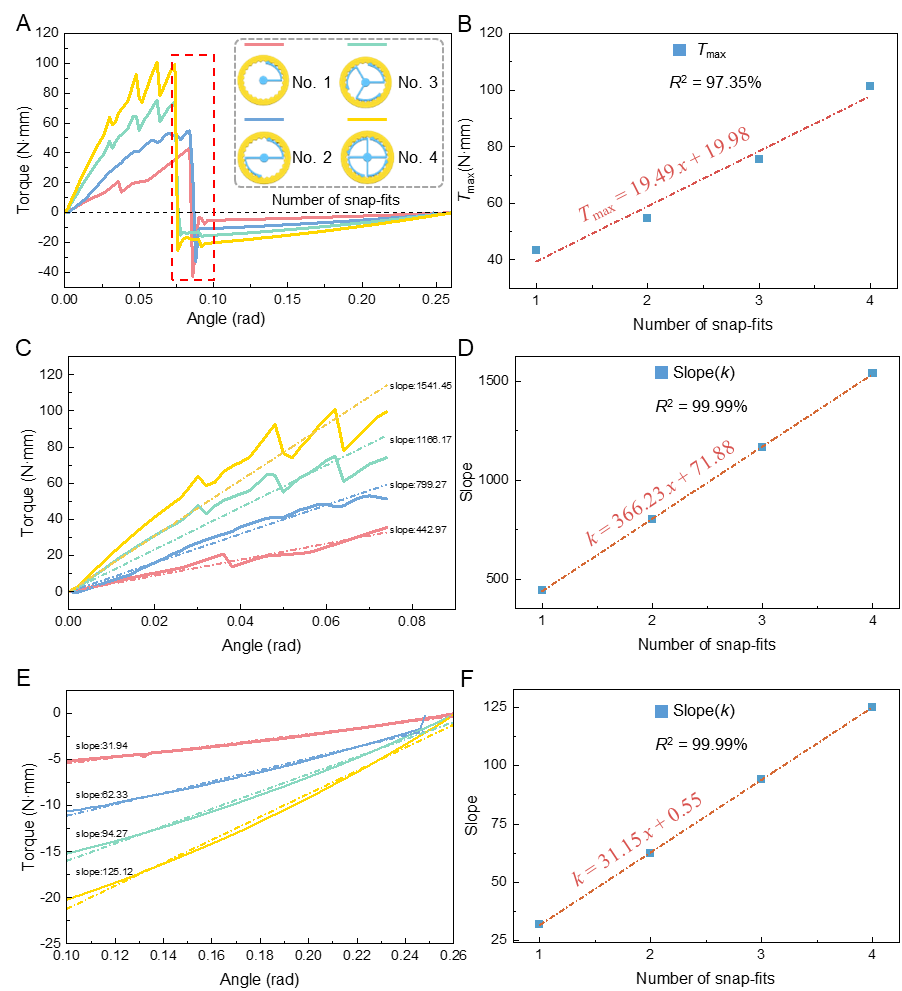
**

**Figure S7 The relationship between torque and the number of snap-fit in the rotationally symmetric snap-fit structures.** (A) It demonstrates the influence of the number of snap-fits on the overall mechanical properties of the structure. The red box marks the steady-state transition region. (B) The relationship between the maximum torque value (T_max_) and the number of snap-fits, along with the results of their linear fitting. (C) The linear fitting situation when the torque is positive. (D) The linear fitting relationship between the slope and the number of snap-fits when the torque is positive. (E) The linear fitting result when the torque is negative. (F) The linear fitting situation between the slope and the number of snap-fits when the torque is negative.

As depicted in **Figure S7A**, it is evident that, except for the region marked by the red box where the steady-state transition occurs, there is a clear linear relationship between torque and the number of snap-fits. A linear fit was performed based on the maximum torque values (*T*_max_) extracted from each structure in **Figure S7A**, and the result is presented in **Figure S7B**. The significant linear correlation between torque and the number of snap-fits can be intuitively observed from the figure. The fitting equation is *T*_max_ = 19.49*x* + 19.98 (where *x* represents the number of snap-fits), and the coefficient of determination, *R*², is as high as 97.4%, close to 1, indicating a good fit.

Furthermore, we conducted linear fits for cases with positive and negative torques separately, with the results shown in **Figures 7C-F**. **Figure S7C** illustrates the scenario with positive torque, from which the slopes under different numbers of snap-fits were obtained, and then the slopes were fitted against the number of snap-fits. It was found that the linearity was extremely high, as shown in **Figure S7D**, with the fitting equation being *k* = 366.23*x* + 71.88 (where *k* is the slope and *x* is the number of snap-fits), and the *R*² value was as high as 99.99%, extremely close to 1. This fully demonstrates a highly linear relationship between the slope and the number of snap-fits when the torque is positive.

Similarly, **Figure S7E** presents the case with negative torque, and the same operation as for positive torque was performed. **Figure S7F** shows the fitting result, with the equation *k* = 31.15*x* + 0.55 (where *k* is the slope and *x* is the number of snap-fits), and its *R*² value was also as high as 99.99%, extremely close to 1. The above analysis strongly confirms the high precision of this linear fit and fully proves that in the rotationally symmetric snap-fit structure (with snap-fit numbers of 1, 2, 3, 4), torque and the number of snap-fit s exhibit a highly linearly correlated characteristic.

Supplementary movies

## Movie S1 Torsion performance test: A torsion tester is used to obtain the torque-angle curve of the sample

## Movie S2 The finite element simulation results of the rotational snap-fit structure stress distribution after deformation

## Movie S3 Applications of 2D rotation-based snap-fit mechanical metamaterials multistable pattern transformations

## Movie S4 Mechanical performance test: conversion mechanism between rotational and translational multistability

## Movie S5 Demonstration of RSMMs-based gripper application multistable gripping operation
